# Trichinellosis in Hospitalized Children and Adults from Western Romania: A 11-Year Retrospective Study

**DOI:** 10.3390/life13040969

**Published:** 2023-04-08

**Authors:** Radu Pavel, Sorin Ursoniu, Maria Alina Lupu, Tudor Rares Olariu

**Affiliations:** 1 Discipline of Parasitology, Department of Infectious Diseases, Victor Babes University of Medicine and Pharmacy, 300041 Timisoara, Romania; 2 Center for Diagnosis and Study of Parasitic Diseases, Victor Babes University of Medicine and Pharmacy, 300041 Timisoara, Romania; 3 Discipline of Epidemiology, Department of Infectious Diseases, Victor Babes University of Medicine and Pharmacy, 300041 Timisoara, Romania; 4 Discipline of Public Health, Department of Functional Sciences, Center for Translational Research and Systems Medicine, Victor Babes University of Medicine and Pharmacy, 300173 Timisoara, Romania; 5 Clinical Laboratory, Institute of Cardiovascular Diseases, 300310 Timisoara, Romania; 6 Patogen Preventia, 300124 Timisoara, Romania; 7 Clinical Laboratory, Municipal Clinical Emergency Hospital, 300079 Timisoara, Romania

**Keywords:** *Trichinella spiralis*, trichinellosis, Western Romania, epidemiology, hospitalized patients

## Abstract

Trichinellosis, a serious and sometimes fatal human disease, is a consequence of consuming raw or improperly cooked meat containing the infective larvae of *Trichinella* spp. The aim of this observational cohort retrospective study is to compare the epidemiological, laboratory, clinical and therapeutic aspects of trichinellosis in children and adults from Western Romania. We investigated the medical records of patients who were diagnosed with trichinellosis and hospitalized between 17 January 2010 and 31 December 2020. One hundred thirty-three patients were identified according to the electronic databases of infectious disease hospitals, located in four counties from Western Romania. A total of 19 patients (14.28%) were children and 114 patients (85.71%) were adults. In children, the most frequent symptoms were digestive in 78.94%, followed by fever in 57.89%, eyelid or facial edema in 57.89% and myalgia in 52.63% of cases, while adults presented mainly myalgia in 87.71%, followed by fever in 77.19%, digestive symptoms in 68.42% and eyelid or facial edema in 66.66% of cases. The source of infection was pork meat products in the majority of patients (89.47%). Our results revealed a general declining trend in infection rates for both children and adults during the studied period. The vast majority of cases were severe and all required hospitalization. Education of the population along with public health strategies should be improved and maintained to fully prevent trichinellosis in Western Romania.

## 1. Introduction

Trichinellosis is a zoonosis with worldwide occurrence caused by parasitic nematodes of the genus *Trichinella* spp. [1]. This serious and sometimes fatal human disease is the consequence of consuming raw or improperly cooked meat containing infective larvae of *Trichinella* spp. [2]. The natural reservoirs of *Trichinella* spp. are wild animals, which are carnivorous and omnivorous. The presence of *Trichinella* spp. in wild and/or domestic animals is not always associated with human infections. The host’s eating habits play an important role in transmission [3]. The most important source of human infection is domestic pigs, especially those raised in free-range pig farms and backyards. However, wild boar meat has played an important role in outbreaks over the past 30 years [4].

The global distribution of *Trichinella* spp. and different cultural food habits are the main factors that favor human infections in industrialized and non-industrialized countries. This zoonosis can be controlled by preventing the transmission of parasites in farms and applying standard animal hygiene procedures correctly in slaughterhouses. *Trichinella* spp. infections derived from wildlife are still endemic in many countries, including fully developed sovereign countries [5].

About 150 years after the discovery of *Trichinella* spp. in the 19th century (Owen,1835), the genus was considered monospecific, and by the early 1970s, biological data suggested that it consists mainly of encrypted parasitic complexes. The presence or absence of a host-derived collagen layer around infected muscle cells leads to the conclusion that the species can be biologically divided into two different groups: encapsulated and non-encapsulated clades [3]. Consequently, two main clades of the *Trichinella* spp. are recognized; one covers a species that is encapsulated in host muscle tissue, and the other does not become encapsulated after muscle cell differentiation [5].

The species and genotypes of the first clade parasitize only mammals (*Trichinella spiralis*; *Trichinella nativa*; *Trichinella britovi*; *Trichinella murrelli*; *Trichinella nelsoni*; *Trichinella patagoniensis*, and *Trichinella* T6; *Trichinella* T8; *Trichinella* T9), whereas of the three species that comprise the second clade, one infects mammals and birds (*Trichinella pseudospiralis*) and two parasitize mammals and reptiles (*Trichinella papuae*; *Trichinella zimbabwensis*) [6]. The individual species or genotypes cannot be distinguished on the basis of morphological characters [7]. With the exception of the existence of capsules and possibly some differences in size, all species and genotypes of *Trichinella* spp. are morphologically indistinguishable in all stages of development [5].

Human infection is divided into two phases: the intestinal and the muscular phases [4]. After digesting the infected meat, during the intestinal phase, larvae are released into the stomach, entering the intestinal mucosa, and by 4–5 days after infection, mature into adult worms [8]. In the systemic and muscular phases, larvae released into the intestinal mucosa migrate into blood vessels and spread throughout the body to reach the striated skeletal muscle cells [8].

There are no accurate data that define the minimal infectious doses that are capable of causing clinical trichinellosis in a person. Meat containing at least one larvae per gram is thought to induce clinical infection in humans and corresponds to about 150 larvae for general consumption. On the other hand, if the number of larvae per muscle biopsy is about 10 per gram, the infection is clinically patent in humans, and if the number of larvae per muscle biopsy is over 100, the infection is severe. The minimum dose of infection can be estimated between 100 and 300 larvae. Over 1000 to 3000 larvae can cause serious diseases [8].

Symptoms are highly correlated with both enteral and parenteral phases [9]. The main clinical symptoms are intestinal diarrhea and abdominal pain, fever, myalgia, myocardial infarction, allergic reactions (especially facial swelling) and encephalitis in the muscular phase [2]. The signs and symptoms of trichinellosis are not typical and misdiagnosis is fairly common [1]. Epidemiological data combined with positive serologic test results and/or identifying larva in a muscle biopsy can confirm the diagnosis [1]. A late diagnosis delays the appropriate treatment, often followed by complications and a more severe course of the disease [10].

Serological methods are widely used to detect animal and human infections [11]. ELISA is the most common diagnostic method for the detection of human and animal *Trichinella* spp. infection. Compared to other serological methods, it is easy to conduct, relatively inexpensive, easy to standardize and can be automated for large-scale tests. ELISA detects *Trichinella* spp. infections with a higher sensitivity than the digestion methods of light-infected animals [12].

Western blot assay can also be applied to detect antibodies against *Trichinella* spp. This method can detect specific antigens of *Trichinella* spp. and distinguish cross-reactive antibodies. This test is not suitable for routine diagnosis because it requires technical expertise, takes time, is cumbersome, costs money, and is often used as an adjunct to confirm other positive-effect serological tests, rather than a routine screening tool for herd samples [12].

The International Commission on Trichinellosis (ICT) does not recommend using laboratory methods to test individual carcasses of animals in slaughter to ensure food safety. In order to detect human infections, conduct epidemiological studies in animals and humans and monitor *Trichinella* spp. infections in pigs, ICT recommends ELISA using excretory/secretory (ES) antigens [11]. Intravital detection of trichinellosis using E-S ELISA tests in experimentally infected pigs was able to determine the IgG antibody concentration compared to excretory secretory antigens of muscle larvae of *T. spiralis* and describe the intensity of *T. spiralis* infection with the same inoculum dose [13].

Histological analysis of muscle tissue may reveal the presence of larvae at different stages of development and collagen capsules (for encapsulated species). This method is more sensitive than trichinoscopy at the first stage of muscle invasion, because larvae are very small and cannot easily be distinguished from muscle fibers [8].

According to Commission Regulation (EU) No. 1375/2015, the criteria for the detection of *Trichinella* spp. larvae in meat are the “magnetic stirring methods for the digestion of pooled samples”, as well as other equivalent methods such as the mechanically assisted/sedimentation method for the digestion of pooled samples, or the automatic digestion method for the digestion of pooled samples up to 35 g [14].

Since 2007, the European Union Reference Laboratory for Parasites (EURLP) has been conducting proficiency tests (PT) for the National Reference Laboratories for Parasites (NRLs) in member states, and public and private laboratories have been conducting official inspections in European and non-EU countries to assess their competence and improve their performance [15]. EURLP organized eight PTs (one per year) to detect *Trichinella* spp. larvae in a meatball panel, containing meat and horse meat mixed with *Trichinella* spp. larvae by molecular methods [15].

The global burden of Trichinella spp. infection is low compared to other foodborne parasitoses [10]. However, in recent years, 5751 cases and five deaths have been reported in 55 countries, and the global disability-adjusted life years (DALY) for trichinellosis was estimated at 76 per billion people per year [2]. Although the global prevalence is difficult to assess, 11 million people worldwide may be infected [16]. *T. spiralis*, a historically imported species from Eastern Asia, is widespread in the Balkan countries (Bulgaria, Romania, Serbia), Lithuania, Poland and Spain [17]. For over a century, trichinellosis has been recognized in most countries in southeastern Europe (Serbia, Montenegro, Croatia, Bosnia and Herzegovina, Bulgaria and Romania) [18].

Today, *T. spiralis* distribution is linked mainly to regions with a high calling for backyard and outdoor pigs, generally corresponding to domestic pigs in countries such as Bosnia–Herzegovina, Bulgaria, Croatia, Estonia, Finland, Germany, Hungary, Ireland, Lithuania, Poland, Romania, Russia, Serbia, Spain and Sweden, while the distribution of *T. spiralis* in wild boar has been mostly reported in regions such as Austria, Bulgaria, Croatia, Czech Republic, Estonia, Finland, France, Germany, Hungary, Lithuania, Netherlands, Poland, Romania, Serbia, Spain and Sweden [6].

From 1986 to 2009, there were 65,818 cases worldwide and Europe accounted for 86% of the cases (56,912). Of the cases reported in Europe, 28,564 (50%) occurred in Romania, mainly between 1990 and 1999. Currently, trichinellosis is a rare but serious disease in the European Union. Almost half of the EU countries have not reported cases of trichinellosis in the last 20 years, including Cyprus, Luxembourg, Malta and Portugal. In the last 16 years (2002–2017), the European Union has recorded 5518 trichinellosis cases, with a decreasing trend [6]. Most human cases were reported from Eastern European countries, and were locally acquired. The decline was mainly due to a marked reduction in the number of cases of trichinellosis reported in Bulgaria and Romania during the same period, which had experienced most of the outbreaks of trichinellosis in previous years [6].

During the last 25 years, the majority of human outbreaks have occurred in Western Romania, where some of the most popular traditional dishes are prepared with raw pork or undercooked pork [18]. According to the European Centre for Disease Prevention and Control (ECDC), over a period of10 years (2007–2016), Romania has reported most of the confirmed cases of trichinellosis in Europe, even though the notification rate and the number of hospitalized patients has been steadily decreasing at a national level [19]. Since 2016, Bulgaria and Italy (recently added in 2020) reported the highest number of confirmed cases [19]. According to the European Food Safety Authority (EFSA) and ECDC, Romania reported 79 *Trichinella* spp. positive findings in domestic pigs and 196 *Trichinella* spp. positive findings in wild boar in 2019 [20].

A recent study revealed a 2% prevalence of *T. spiralis* infection in blood donors from Western Romania [21]. This study aimed to assess the epidemiological, laboratory, clinical and therapeutic aspects of trichinellosis in children and adults from Western Romania, a well-known endemic region.

## 2. Materials and Methods

An observational cohort retrospective study was conducted between 17 January 2010 and 31 December 2020. We decided to expand the study period and geographical area for the previous published five year (2012–2016) retrospective study, performed in 3 counties [22]. Electronic databases of infectious disease hospitals, located in 4 counties from Western Romania (Arad, Caraș-Severin, Hunedoara and Timiș), with a total population of 1,828,313 [23], were investigated. One hundred thirty-three patients diagnosed with trichinellosis were identified. Based on patients’ medical records, the diagnosis was established by corroborating the results with those provided by the history of eating raw/uncooked meat, clinical signs, serologic test results for *Trichinella*-specific antibodies, patients’ muscle biopsy results and epidemiological links (exposure to a common source of contaminated meat) [8].

From the patients’ medical records, we extracted data regarding age, gender, area of residence, clinical signs, date of hospital admission, hospitalization length of stay, evolution, source of infection, routine laboratory investigation results (leukocyte and eosinophil count, erythrocyte sedimentation rate), serologic test results for *Trichinella*-specific antibodies and specific medical treatment.

Study participants were grouped into children and adults based on their age: 1–17 years and over 18 years, respectively. Within each group, individuals were further divided as follows: (i) children:1–9 years and 10–17 years; (ii) adults: 18–28 years, 29–39 years, 40–49 years, 50–59 years and over 60 years.

Data were compiled in a Microsoft Excel database, version 2011 (Microsoft Corp., Redmond, WA, USA). Statistical analyses were performed using Epi Info Version 7.2 (CDC, Atlanta, GA, USA) and Stata 16.1 (StataCorp, College Station, TX, USA). Data are presented as percentage (number), mean ± standard deviation (SD), median and interquartile range (IQR), odds ratios (OR) and 95% confidence intervals (CI). Bartlett’s test was used to evaluate thenormal distribution of variables, Student’s *t*-test for differences between means in the studied groups, Mantel–Haenszel chi-square test or two-tailed Fisher’s exact test, as appropriate, to compare frequency distribution between two groups. Linear regression analysis was performed to assess trends over time for infection rates in children and adults. A probability level of *p* < 0.05 was considered to indicate statistical significance.

## 3. Results

Between 2010 and 2020, 133 patients were diagnosed with trichinellosis and hospitalized in Western Romania. They were aged between 1 and 78 years (mean age = 36.75 ± 16.87 years): 55.64% (74/133) were males and 69.92% (93/133) were residents of rural areas. Of the 19 (14.28%) children aged 1–17 years (mean age = 9.26 ± 5.86 years), 36.84% (7/19) were males and 73.68% (14/19) were inhabitants of rural areas. A total of 114 patients (85.71%) were adults, aged 18–78 years (mean age = 41.33 ± 13.37 years); 58.77% (67/114) were males and 69.30% (79/114) were inhabitants of rural areas (Table 1).

**Figure 1:**
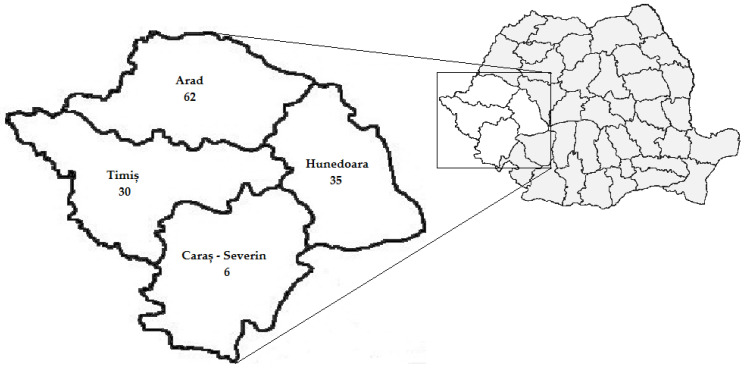
Distribution of trichinellosis cases in Western Romania by county of residence, 2010–2020.

The study participants were residents of Arad 46.62% (62/133), Hunedoara 26.32% (35/133), Timiș 22.56% (30/133) and Caraș-Severin 4.51% (6/133) counties (Figure 1).

**Figure 2:**
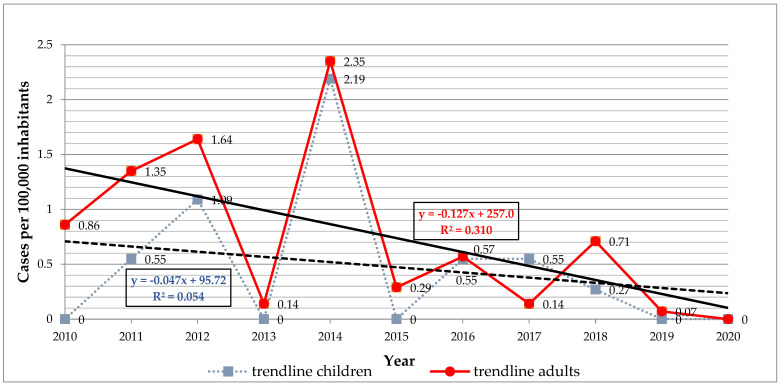
Distribution of trichinellosis cases in children and adults from Western Romania between 2010 and 2020.

The infection rate (cases per 100,000 inhabitants) for the studied period was 14.4 in Arad, 8.36 in Hunedoara, 4.39 in Timiș and 2.03 in Caraș-Severin. The mean annual infection rate was 0.47 in the children (from 0.0 to 2.19) and 0.73 in the adults (from 0.0 to 2.35) (Figure 2). Our results revealed a general declining trend in infection rates with no significant differences, for both children (*r* = −0.23, *p* = 0.48) and adults (*r* = −0.55, *p* = 0.07) (Figure 2).

In the children, the number of *Trichinella* spp. infection cases was higher in the age group of 10–17 years at 52.63% (10/19) compared to the age group of 1–9 years at 47.36% (9/19). In adults, the highest number of cases occurred in the age group of 29–39 years at 34.21% (39/114) and the lowest was in those aged over 60 years at 10.52% (12/114) (Table 1).

When the data were analyzed according to gender and area of residence, no significant difference between the children and adults was observed (*p* = 0.07 and *p* = 0.70, respectively) (Table 1).

Most cases of trichinellosis were recorded in the winter season: 57.89% (11/19) in children and 64.91% (74/114) in adults. No significant difference between the two groups was observed according to seasons (*p* = 0.79) (Table 1).

Overall, the incubation period ranged from 1 to 30 days (mean = 8.42 ± 5.89 days), with no significant difference between the children (range: 2–21 days, mean = 7.21 ± 4.44 days) and adults (range: 1–30 days, mean = 8.63 ± 6.09 days) (*p* = 0.23).

In the children, the most frequent symptoms included digestive symptoms in 78.94% (15/19), fever in 57.89% (11/19), eyelid or facial edema in 57.89% (11/19) and myalgia in 52.63% (10/19) of cases (Table 1). The adults mainly presented myalgia in 87.71% (100/114), followed by fever in 77.19% (88/114), digestive symptoms in 68.42% (78/114) and eyelid or facial edema in 66.66% (76/114) of cases. Headache, asthenia, conjunctival and subungual hemorrhages, skin rash, chills and excessive sweating were also described (Table 1). No significant difference regarding clinical signs and symptoms between children and adults was observed (*p* = 0.31) (Table 1).

When the data were analyzed according to laboratory findings, no significant difference was found between the children and adults regarding eosinophil count, leukocyte count and erythrocyte sedimentation rate (Table 2). Of the 133 patients diagnosed with trichinellosis, 17.29% (23) of patients were tested for *Trichinella* spp. specific antibodies and 65.22% (15/23) tested positive. No subsequent serological investigations or molecular tests for *Trichinella* spp. identification were performed.

All children and adults received specific antihelminthic treatment (albendazole or mebendazole). The patients with marked symptomatology received corticosteroids associated with anthelminthic treatment. Albendazole was the treatment of choice for 15.79% (3/19) of children and 27.19% (31/114) of adults, and albendazole was associated with corticotherapy for 52.63% (10/19) of children and 56.14% (64/114) of adults, with no significant difference between groups (*p* = 0.75).Mebendazole was received by 15.79% (3/19) of children, while mebendazolewasassociated with corticotherapy in 15.79% (3/19) of children and 16.66% (19/114) of adults, and a significant difference between these twogroups was observed (*p* < 0.05) (Table 3).

The length of hospital stay varied between 2 and 21 days (mean = 7.21 ± 4.44 days) in the children and between 1 and 17 days (mean = 7.46 ± 3.41 days) in the adults, with no significant difference (*p* = 0.81) (Table 3). There were no significant differences between means of hospital stay regarding treatment (antihelminthic drugs alone versus association of antihelminthics and corticotherapy) in children (*p* = 0.15) and adults (*p* = 0.74), respectively.

Clinical evolution was moderately severe in 36.84% (7/19) of children and 32.46% (37/114) of adults. A severe course of the disease was noted in 52.63% (10/19) of children and 57.02% (65/114) of adults (Table 3). Most patients (99.74%; 130/133) fully recovered after infection. However, one male patient aged 61 years presented right hemiplegia at discharge and two patients, aged 68 and 61 years, died.

The source of infection was pork meat products in 89.47% (119/133) of patients, wild boar meat products in 5.26% (7/133) of patients and a combination of pork and wild boar meat products in 0.75% (1/133) of patients. In six patients (4.51%), the source of infection was not mentioned in the electronic databases and/or patient charts. Statistical analyses performed on the patients who met the epidemiological criteria of exposure to a common source of infection showed a significant association with pork meat products as the source of infection (OR = 16.29; 95% CI: 4.63–57.32; *p* < 0.001). The epidemiological criteria for exposure to a common source of infection were met by 87.22% (116/133) of patients and they were part of 18 different family outbreaks. Patients who did not meet the epidemiological criteria, 12.78% (17/133), were sporadic cases.

## 4. Discussion

Our results showed that 133 patients diagnosed with trichinellosis required hospitalization in Western Romania. This is a significant downward trend number compared to the 1344 patients diagnosed with *Trichinella* spp. infection between 1996 and 2005 [24] in the same region. This decreasing trend may be explained by better awareness of infection, increased control measures at slaughterhouses and improvements in pig-rearing practices [25].

The number of *Trichinella*-infected children was lower compared to adults in our study and this is consistent with previous reports from France [26] and Italy [17]. In the trichinellosis outbreak reported in Serbia in 2017, children were more infected compared to adults [26]. Children may be considered infected by parents’ irresponsibility [27]. In the present study, the annual average incidence of trichinellosis was higher in adults compared to children, in contrast to previously reported results [24]. Our observation showed that the annual average incidence for the studied period was lower compared to the incidence reported by Neghina R et al. [24] and the declining trend in infection rates continued.

The patients included in our study were represented mostly by adult males, contrary to other reported observations [24,28,29] and by female children, similar to results published by Khurana S et al. [30]. Patients residing in rural areas were most representative in our study, similar to other observations [31], but in contrast with other studies [24,32].

We found that winter was the season with the highest number of trichinellosis cases, as described in other studies [10,18,24,32,33]. In January and February, various pork products are consumed during Christmas and the wild boar hunting season [6]. Pigs are slaughtered in the winter season and traditional products (raw or uncooked) are mainly consumed during holidays such as Christmas and New Year’s Eve, and during the upcoming months of the spring [34].

The incubation period depends on the severity of the disease: it may last one week in severe forms, two weeks in moderate forms, and three to four weeks in benign and/or abortive forms [1,8]. Our findings showed no significant difference in the mean for the incubation period between children and adults. It has been noted that the incubation period is shorter in severe forms of trichinellosis [8] and the results of the present study confirmed this observation, since all patients required hospitalization. The average incubation period was similar to that reported in a previous retrospective study, with 7.6 days [22], but shorter than the one reported by Ozdemir D et al., with 14.3 days for children and 15.3 for adults [35].

The signs and symptoms of trichinellosis are similar in children and adults [8]. However, the anecdotal evidence published so far suggests that children have mild symptoms and a milder course of disease compared to adults due to a lower infecting dose and a lower allergic response to *Trichinella* spp. antigens [35]. Myalgia is related to the severity of the illness [1] and our observations revealed that myalgia occurred in a similar percentage to previous studies in children and adults [24] but lower compared to other reported results for children [30]. We had no information regarding the precise amount of meat consumed by the patients, so we could not evaluate the worm burden to which they were exposed. Therefore, we cannot explain the differences. The overall clinical picture of our patients diagnosed with trichinellosis was consistent with the pattern of symptomatology reported in the literature (digestive symptoms, fever and facial edema) [8,17,26,35].

Complications have been observed mainly in severe cases, particularly in the elderly, and a positive correlation has been reported between age and severity of complications [8]. Brain damage can result in diffuse encephalopathy or focal signs, transient hemiparesis or hemiplegia [1] and death from trichinellosis, even rare, is reported in people aged >65 years [1]. In our study, one patient remained with a motor deficit after discharge and two died due to neurological complications.

In the present study, laboratory investigation findings (eosinophil counts; leukocyte counts; erythrocyte sedimentation rate) showed higher values in adults compared to children, with no significant difference. Evaluating the presence of specific antibodies has great diagnostic value [4], but negative results are frequently reported during the first week of the disease and seroconversion is observed when the patient is beginning to recover [29]. Furthermore, antibodies are usually not detected when clinical symptoms occur, and in the first few days of febrile phase, the serodiagnosis is sometimes negative. In this case, it is desirable to order a second test a few days later [1].

A previously conducted cross-sectional study that investigated sera for anti-*T. spiralis* antibodies in 1347 consecutive blood donors residing in Western Romania showed a seroprevalence of 2%. The *Trichinella* spp. seropositive subjects were asymptomatic individuals who were not previously diagnosed or treated for this zoonosis [21]. Of the 23 patients tested for *Trichinella* spp. antibodies in our study, 65.22% (15) had positive results. The undetectable antibodies in patients diagnosed with trichinellosis may be explained by the ingestion of a low number of infectious larvae [17].

As a result, the number of infections in many countries may be under-reported due to a lack of appropriate serological tests [8]. The lack of information in a country does not mean that these zoonotic infections do not exist, but rather may reflect the lack of laboratory investigations [8,36]. In many cases, there may be an existing infection that is not reported and cannot be recognized [36].

Early diagnosis of trichinellosis leads to early initiation of antihelminthic treatment (with albendazole or mebendazole) against adult worms in the intestine, migrating larvae and larvae in the muscles before the development of the collagen capsule [35]. Most experts recommend the association of diffusible antihelminthics and corticosteroids to prevent the occurrence of complications [8,36]. The decision to recommend post-exposure prevention for all persons exposed to meat that contains viable *Trichinella* spp. larvae should not be based on symptoms, but on potential exposure (because early symptoms of trichinellosis are not specific) [37]. Therefore, in cases of clusters of similar clinical patterns of infections, it is important to obtain a detailed history of food consumption as soon as possible [38]. In this study, all patients received specific antihelminthic treatment (mebendazole or albendazole), associated with or without corticosteroids, similar to other prior observations [22,24].

According to the Commission Regulation No. 2015/1375 [14], all *Trichinella* spp. susceptible animals intended for human consumption in the EU should be tested for *Trichinella* spp. [39]. Domestic pigs and hunted wild boar that are not placed on the EU market but are considered for their own consumption are absolved from the above-mentioned regulation [39]. In Europe, wild boar meat has been responsible for many reported human outbreaks and is considered an important source of human trichinellosis, along with pork meat. Recreational hunting and consumption of undercooked wild boar meat have increased the risk of acquiring trichinellosis, especially for hunters, their families and friends [2,40,41,42]. In the present study, we found that pork and wild boar-consumed meat products came from non-commercial sources or privately raised animals. In Romania, the prevalence of *Trichinella* spp. was found to be 8.9% in home-slaughtered pigs and 8.6% in wild boars [43] and this may explain the ongoing infection in Romanian children and adults. Our findings regarding the source of infection, represented by pork meat and wild boar meat products, were similar with those described by other authors [30,31,33,37,38,44,45].

This study has limitations due to the relatively low number of cases and its retrospective design. Our findings are based on the information collected from patients’ medical records. Therefore, results presented in this study reflect only the information available in patient charts and hospital electronic databases. However, this research was performed in infectious disease reference hospitals, located in an extended area (four counties) of Romania and revealed that trichinellosis is still a problem in this European Region.

## 5. Conclusions

The infection rates of trichinellosis for the studied period showed a general declining trend in Western Romania. However, the vast majority of trichinellosis cases were represented by severe cases and all required hospitalization.

Complications are usually observed in severe cases, but are also described in moderate cases, including persons who have been treated improperly or have started treatment too late [8]. Life-threatening encephalitis and myocarditis often occur simultaneously. Other complications can occur such as thromboembolic disease, ocular lesions with disturbances in microcirculation, neuromuscular disturbances, digestive complications [8] and even secondary infections (bronchopneumonia and sepsis) [4].

It is important to note that many severe cases of trichinellosis or those with complications may require lengthy antiparasitic treatment. It should be remembered that *T. spiralis* larvae are resistant to mebendazole treatment and can live up to 30 years in muscle cells once encapsulation has begun in humans. Mebendazole and probably also albendazole are inefficient at killing encapsulating larvae in humans [46].

Therefore, considering the importance of the complications that may appear during the evolution of the disease, and the fact that some cases may end with the death of the patient, eating tested meat in accordance with Commission Regulation 2015/1375 and testing by ISO 18743:2015 is of great importance.

ISO 18743 is the first international standard in food microbiology for the detection of parasites and may be considered a document that has an impact on the food industry. In line with the Commission’s Regulation 2015/1375, it also represents an opportunity to harmonize official control with international regulations to ensure the safety of meat and facilitate import/export trade [47].

International standards may be of great importance in supporting policy makers, especially in countries without expertise and/or resources or legislation on specific issues such as food safety, thereby improving access to the global market [47].

Most of the patients were residents of rural areas and a significant number of patients were part of different family outbreaks. Education of the population, adapted to its level of understanding regarding their food habits, in particular, undercooked, incorrectly processed meat or raw meat consumption which play a key role as risk factors for trichinellosis, alongside public health strategies, should be improved and maintained to fully prevent trichinellosis in Western Romania.

## Figures and Tables

**Table 1:** Demographics and clinical findings in children and adults diagnosed and hospitalized with trichinellosis in Western Romania between 2010 and 2020.

	Variables	No. Patients	Children (*n* = 19) No. (%)	Adults (*n* = 114) No. (%)	*p*-Value
Age groups (years)	1–9	9	9 (47.36)	-	NA
	10–17	10	10 (52.63)	-	
	18–28	19	-	19 (16.66)	
	29–39	39	-	39 (34.21)	
	40–49	27	-	27 (23.68)	
	50–59	17	-	17 (14.91)	
	≥60	12	-	12 (10.52)	
Gender	Male	74	7 (36.84)	67 (58.77)	0.07 ^1^
	Female	59	12 (63.16)	47 (41.23)	
Residence area	Urban	40	5 (26.32)	35 (30.70)	0.70 ^1^
	Rural	93	14 (73.68)	79 (69.3)	
Season of acquiring the infection	Spring	8	1 (5.26)	7 (6.14)	0.79 ^1^
	Summer	2	0 (0)	2 (1.75)	
	Autumn	38	7 (36.84)	31 (27.19)	
	Winter	85	11 (57.89)	74 (64.91)	
Clinical signs	Myalgia	110	10 (52.63)	100 (87.72)	0.31 ^1^
	Fever	99	11 (57.89)	88 (77.19)	
	Digestive symptoms (nausea, abdominal pain, diarrhea)	93	15 (78.95)	78 (68.42)	
	Eyelid or facial edema	87	11 (57.89)	76 (66.67)	
	Asthenia	65	5 (26.32)	60 (52.63)	
	Headache	53	8 (42.11)	45 (39.47)	
	Rash	25	4 (21.05)	21 (18.42)	
	Chills	19	0 (0)	19 (16.67)	
	Conjunctival and subungual hemorrhages	17	4 (21.05)	13 (11.4)	
	Excessive sweating	8	0 (0)	8 (7.02)	

NA, not applicable. ^1^ Mantel–Haenszel chi-square test. No., number of trichinellosis cases.

**Table 2:** Laboratory findings in children and adults with trichinellosis from Western Romania between 2010 and 2020.

	Variable	No. Patients	Children (*n* = 19) No. (%)	Adults (*n* = 114) No. (%)	*p*-Value
Eosinophil counts (%)	(21.06 ± 14.84) ^1^	-	(15.8 ± 13.98) ^1^	(21.94 ± 14.85) ^1^	0.09 ^2^
	<5	13	3 (15.79)	10 (8.77)	0.39 ^3^
	>5	120	16 (84.21)	104 (91.23)	
Leukocyte counts (cells/mm^3^)	10,700 (7800;13,850) ^4^	-	9310 (6220; 12,800) ^4^	11,110 (8130; 14,220) ^4^	0.08 ^5^
	<10,000	64	12 (63.16)	52 (45.61)	0.15 ^6^
	>10,000	69	7 (36.84)	62 (54.39)	
Erythrocyte sedimentation rate (mm/h)	(17.60 ± 11.42) ^1^	-	(15.73 ± 9.30) ^1^	(17.91 ± 11.74) ^1^	0.37 ^2^
	<10	39	8 (42.11)	31 (27.19)	0.18 ^6^
	>10	94	11 (57.89)	83 (72.81)	

^1^ Mean ± SD. ^2^ Student’s *t*-test. ^3^ Fisher’s exact test. ^4^ Median (interquartile range). ^5^ Mann-Whitney test. ^6^ Mantel–Haenszel chi-square test. No., number of trichinellosis cases.

**Table 3:** Treatment, length of hospital stay and course of disease in children and adults with trichinellosis from Western Romania between 2010 and 2020.

	Variable	No. Patients	Children (*n* = 19) No. (%)	Adults (*n* = 114) No. (%)	*p*-Value
Treatment	Albendazole	34	3 (15.79)	31 (27.19)	0.75 ^1^
	Albendazole and corticotherapy	74	10 (52.63)	64 (56.14)	
	Mebendazole	3	3 (15.79)	0 (0)	<0.05 ^1^
	Mebendazole and corticotherapy	22	3 (15.79)	19 (16.66)	
Length of hospital stay (days)	(7.42 ± 3.55) ^2^		(7.21 ± 4.44) ^2^	(7.46 ± 3.41) ^2^	0.81 ^3^
	1–7	78	12 (63.16)	66 (57.89)	0.90 ^4^
	8–14	48	6 (31.58)	42 (36.84)	
	15–21	7	1 (5.26)	6 (5.26)	
Course of disease	Severe	75	10 (52.63)	65 (57.02)	0.94 ^3^
	Moderately severe	44	7 (36.84)	37 (32.46)	
	Benign	9	1 (5.26)	8 (7.02)	
	Abortive	5	1 (5.26)	4 (3.51)	

^1^ Fisher’s exact test. ^2^ Mean ± SD. ^3^ Student’s *t*-test. ^4^ Mantel–Haenszel chi-square test. No., number of trichinellosis cases.

## Data Availability

The data are available on request.
